# Health Insurance and Out-Of-Pocket Expenditure on Health and Medicine: Heterogeneities along Income

**DOI:** 10.3389/fphar.2021.638035

**Published:** 2021-04-30

**Authors:** Mohammed Khaled Al-Hanawi, Martin Limbikani Mwale, Ameerah M. N. Qattan

**Affiliations:** ^1^ Department of Health Services and Hospital Administration, Faculty of Economics and Administration, King Abdulaziz University, Jeddah, Saudi Arabia; ^2^ Department of Economics, Faculty of Economic and Management Sciences, Stellenbosch University, Cape Town, South Africa

**Keywords:** insurance, medicines, out-of-pocket expenditure, health, saudi arabia (KSA), income

## Abstract

**Background:** Achieving universal health coverage is an important objective enshrined in the 2015 global Sustainable Development Goals. However, the rising cost of healthcare remains an obstacle to the attainment of the universal health coverage. Health insurance is considered an option to reduce out-of-pocket (OOP) expenditure on health and medicine. Nevertheless, the relationship between insurance and the OOP along welfare distributions is not well understood. This study investigates the heterogeneous association between health insurance and OOP expenditure on health and medicine, along income, using data from the Kingdom of Saudi Arabia.

**Methods:** This study used data of 8655 individuals drawn from the Saudi Family Health Survey conducted in 2018. The study adopts Tobit models to account for possible corner solution due to individuals with zero expenditure on health. We minimize the confounding effects of non-random selection into the insurance program by estimating the Tobit equations on a sample weighted by inverse propensity scores of insurance participation. In addition, we test whether the health insurance differently relates to OOP on health and medicine amongst people with access to free medical care as opposed to those without this privilege. The study estimates separate models for OOP expenditure on health and on medicines.

**Results:** Health insurance reduces OOP expenditure on health by 2.0% and OOP expenditure on medicine by 2.4% amongst the general population while increasing the OOP expenditure on health by 0.2% and OOP expenditure on medicine by 0.2%, once income of the insured rises. The relationship between the insurance and OOP expenditure is robust only amongst the citizens, a sub-sample that also has access to free public healthcare. Specifically, the insurance reduces OOP expenditure on health by 3.6% and OOP on medicine by 5.2% and increases OOP expenditure on health by 0.4% and OOP expenditure on medicine by 0.5% once income of the insured increases amongst Saudi citizens. In addition, targeting medicines can lead to greater changes in OOP. The relationship between insurance and OOP is stronger for medicine relative to that observed on health expenditure.

**Conclusion:** Our findings suggest that insurance induces different effects along the income spectrum. Hence, policy needs to be aware of the possible welfare distribution impacts of upscaling or downscaling the coverage of insurance amongst the populations, while pursuing universal healthcare coverage.

## Introduction

The economics textbook expectation is that health insurance, provided at actuarially fair price with full coverage, induces risk-averse individuals into participation, with the quest of reducing unanticipated financial risk (Wagstaff and Lindelow, 2008). By implication, health insurance should, therefore, reduce out-of-pocket (OOP) expenditure on health. However, empirical evidence reveals different outcomes. In some contexts health insurance indeed reduces OOP expenditure (Ahmed et al., 2020; Harish et al., 2020; Sriram and Khan, 2020) while increasing the OOP expenditure in alternative settings (Li et al., 2020; Okoroh et al., 2020; Ying and Chang, 2020). These contradictions invite the question whether policy should expand or contract the provision and coverage of health insurance in the best interest of citizens. The question is more relevant now than before since countries embarked on increasing universal Health Coverage (UHC) upon the adoption of the Sustainable Development Goals in 2015.

Several countries established health insurance as one of the mechanisms for attaining UHC (Buttorff et al., 2015; Fang et al., 2019; Matsushima et al., 2020). However, the effectiveness of the insurance in increasing proper healthcare access for all depends on the behavior responses and health expenditure implications of different subsections of the targeted populations (Kraft and VD Schulenburg, 1986). For instance, if health insurance compels healthcare providers to shift the patient’s demand curve to the right due to asymmetric information, the affluent would increase OOP expenditure. The poor might only reduce OOP as the insurance covers their medical cost while having less income to attain the supplier induced demand services. This makes understanding of the different effects of health insurance along welfare spectra vital for UHC attainment. This study, therefore, aims to estimate the heterogeneous effects of health insurance, along income, on OOP expenditure. The study uses data from the Kingdom of Saudi Arabia (KSA), one of the countries that has been implementing a health insurance scheme for about two decades.

Saudi Arabia is a suitable case study to understand the relationship between health insurance and OOP expenditure. Despite being a high-income country, the KSA (just like other countries in the Arabian Gulf region) has some low-income healthcare attributes which make its context applicable to results from neither high- nor low-income effects of insurance literature. Firstly, the country provides free healthcare to its citizens through public health facilities which increases pressure on public healthcare provision (Al-Hanawi et al., 2018a). Secondly, about 56% of its workforce are expatriates who do not access the free healthcare and therefore rely on insurance and OOP expenditure (Alkhamis, 2018). Therefore, results from the KSA could reveal not only the heterogeneous relationship between insurance and OOP expenditure but also how this relationship can be modified under strained free healthcare system. To the best of our knowledge, this study is the first to be conducted in an oil dependent country that finances healthcare using the finite natural resource (Al-Hanawi et al., 2018b). Considering the potential threat of continued fall in oil prices, countries beyond the KSA that also finance healthcare using the natural resource, would benefit from our results, whether to consider insurance as an effective means of insuring sustainability of healthcare financing while pursuing universal health coverage.

This study contributes to prevailing debate on the relationship between health insurance and OOP expenditure on health by not just examining the direction but also how income mediates this relationship of interest. If the relationship differs based on the level of income, then, this study provides the much-needed direction that policy can choose strategies from while being fully aware of the distribution impacts of the course of action. In addition, we demonstrate whether the analysis of the relationship between health insurance and healthcare access should account for welfare distribution to provide reliable estimates. Considering that medicines expenditure forms the largest component of OOP expenditure (Wirtz et al., 2012; Zullo et al., 2017; Ghosh et al., 2019), this study further examines a particular relationship between health insurance and OOP expenditure on medicine, besides the general OOP expenditure on health outcome.

### Healthcare Access and Health Insurance in Saudi Arabia

The KSA provides free access to healthcare services through the public health facilities to both Saudis and non-Saudis working in the government sectors. Furthermore, the KSA provides free healthcare services to the general public, which exorbitantly raises the cost of financing healthcare in the kingdom exacerbated by the rapid demographic changes, an aging population, changing disease pattern, and increased prices of medical technology (Almalki et al., 2011). Public health provision in the KSA is of high quality. However, it has faced efficiency challenges due to the overwhelmingly large number of people that it caters for (Al-Harajin et al., 2019). These public healthcare bottlenecks lead to increased OOP expenditure amongst some citizens. Unsurprisingly, most private healthcare services are provided to Saudis who are eligible for free healthcare services through the public sector (Walston et al., 2008). Besides, the kingdom has over 75% of private sector employees as expatriates, accounting for 56% of the gross Saudi workforce (Alkhamis, 2018). These attributes pose a further strain on the KSA healthcare resource envelope.

In response, the Saudi government enacted the Cooperative Health Insurance Law in 1999 that established a mandatory health insurance scheme for private sector employees in 2002 (Al-Sharqi and Abdullah, 2013; Alkhamis et al., 2014). The scheme aimed to force private sector employers to cover healthcare costs for their employees (Saudi and non-Saudi) in a quest to relieve pressure off public health services by pushing the private sector employees to private healthcare providers (Al-Hanawi et al., 2018a). Under the scheme, the insured obtain healthcare services from private healthcare facilities, and the employers pay a full amount for the premium that, however, differentiates coverage packages across employees’ profiles (Alkhamis, 2018). It is worth noting that the general population including the public sector employees are also allowed to access private health facilities and pay out-of-pocket or purchase private health insurance packages to safeguard their income and wealth against unanticipated health shocks from illnesses. Currently, the scheme has 27 insurance companies catering for 11 million beneficiaries, that access private healthcare (Rahman, 2020).

The Council for the Cooperative Health Insurance (CCHI) coordinates and manages the provision of health insurance in the KSA. The CCHI determines all minimum healthcare needs and treatments that are provided for under a unified insurance benefit package (Alkhamis, 2018). While some top-ranking employees enjoy comprehensive coverage from their companies and institutions, some workers have partial coverage. Those under partial coverage incur co-payments for their medical bills because the unified package remains insufficient (Aidam et al., 2016).

Against this backdrop, both citizens and expatriates in the KSA remain at risk of increased OOP expenditure. The question remains whether, besides easing pressure on public facilities, policy should expand access to health insurance to move toward equitable access to healthcare. The policy stance also remains a dilemma particularly because of the missing consensus on the direction of the relationship between health insurance and OOP expenditure in the healthcare access literature. In this study, we uncover the possible heterogeneous direction of the relationship; how different levels of income mediate the relationship between health insurance and OOP expenditure.

### Conceptual Framework

The economic question we set out to answer is: “How does participation in health insurance relate to OOP expenditure on health, along different income levels?” Part of the answer can be found in literature (Gnawali et al., 2009; Nguyen et al., 2011; Zimmer, 2011; Roos and Schut, 2012; Cantarero-Prieto et al., 2017; Dong et al., 2018) that examines the effects of health insurance on healthcare utilization. While available literature deals with whether health insurance leads to either positive or negative or no effects at all, we depart from that focus to examine the possible outcomes subject to the income levels of the insured. Our hypothesis is that individuals that subscribe to health insurance should experience a reduction in OOP expenditure and increase consumption of prescribed care conditional on income that allows them to join this high level of healthcare.

**FIGURE 1:**
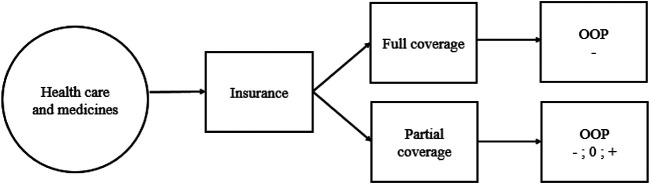
Conceptual framework for the economic decision to incur out of pocket expenditure.

Theoretically (as shown in Figure 1), an individual anticipating financial risk due to uncertain healthcare costs should purchase health insurance based on incentives that their health insurance package provides consistent with their risk. A more generous package, that is actuarially fair and offers full coverage, should attract risk-averse individuals (Wagstaff and Lindelow, 2008). The perceived benefit is the comfort that whether one is ill or not, their finances are secured since insurance takes full care of the medical costs. These are individuals who will most likely reduce OOP expenditure once they are insured and could have no desire to use the savings in higher level healthcare consumption.

In the event of non-generous, partial coverage, risk perverse individuals may purchase health insurance. However, unlike the risk averse, these individuals’ behavior may not be predicted. If they use the savings from reduced expenditure from health in alternative means, OOP will reduce (negative sign). Alternatively, they may still incur OOP only to a level offsetting the saving from usage of health insurance (zero change). This will result in no significant net change in OOP. A third option is where these individuals demand expensive care which they cannot afford in the absence of health insurance (positive sign). The overall effects of health insurance on OOP become positive.

In practice, the increased demand for expensive healthcare could emerge from health-seeking effects of health insurance combined with opportunistic behavior of providers with asymmetric information (Ying and Chang, 2020). Insurance increases number of medical visits that individuals make to healthcare providers (Al-Hanawi et al., 2020). Healthcare providers use their superior asymmetric information advantage to prescribe medicine that is not covered by the insurance (Bernal et al., 2017). Consequently, the insured spend more resources on medical care than they would without the health insurance. Nevertheless, the insured would do so, only when they have the resources required to procure the prescription. Therefore, the relationship between health insurance and OOP expenditure conditional on income levels remains an empirical question which this study intends to answer using data from the KSA.

## Materials and Methods

### Data and Variables

This study draws secondary data from the 2018 Family Health Survey (FHS) conducted by the General Authority for Statistics (GaStat) in the KSA (GASTAT, 2018). The FHS is the first collaborative stage between GaStat and several entities in the health sector in the Kingdom such as the Ministry of Health, the Saudi Health Council, as well as the private and academic sectors. The FHS is a field survey conducted every three years by GaStat and it falls under the classification of education and health statistics. The FHS collects information by visiting a representative sample of the population across all 13 administrative regions in the KSA. An important element to our study is that the health status section of the survey includes a question on whether an individual is covered by health insurance or not. Further, the respondents also report on OOP health expenditures that they incurred. A follow up question asks the amount of OOP that the individual spent, particularly on medicine. The survey also contains rich information on demographic and socioeconomic status.

Given the richness in health-related information and representativeness, the FHS is ideal for examining the heterogeneous relationship between health insurance and OOP along different levels of income. The FHS collected a total sample of 15,265 responses randomly selected across the 13 administrative regions of the KSA. This study limits the analysis to respondents who have complete information on all the variables of interest. Therefore, this study’s analysis is based on a sample of 8,655 respondents after dropping those with non-responses to healthcare related questions and covariates.

The main outcome variables for this study are OOP expenditure on health and OOP expenditure on medicine. Table 1 provides the specific definitions, means and standard deviations of the outcome variables and all other independent variables used in the study. OOP is measured as a continuous variable in Saudi Riyal (SR) (1 US$ = 3.75 SR). On average, OOP expenditure on medicine forms 53% of the total OOP expenditure on health in the sample. The main independent variable used in this study is health insurance. Health insurance is captured as a binary variable with 1 if respondent is covered by health insurance and 0 if otherwise. In our selected sample, 30% of the respondents have health insurance. The second independent variable used in this study is income. The income is a continuous variable and measured in SR.

**TABLE 1:** Variable definition, specification, and summary statistics (*N* = 8655).

Variable	Definition	Mean	Std. Dev
OOP expenditure on health	Continuous	747.018	1194.665
OOP expenditure on medicine	Continuous	394.113	889.001
Health insurance	Dummy (1 insured; 0 uninsured)	0.298	0.457
Monthly income	Continuous	11,443.920	10,639.740
Nationality	Dummy (1 saudi; 0 non-saudi)	0.770	0.421
Age	Continuous	36.514	21.090
Gender	Dummy (1 male; 0 female)	0.525	0.499
Marital status	Dummy (1 married; 0 unmarried)	0.590	0.492
Below primary school	Dummy (1 below primary school; 0 otherwise)	0.200	0.400
Primary school	Dummy (1 primary school; 0 otherwise)	0.139	0.346
Intermediate school	Dummy (1 intermediate school; 0 otherwise)	0.157	0.364
Secondary school	Dummy (1 secondary school; 0 otherwise)	0.308	0.462
Higher education	Dummy (1 higher education; 0 otherwise)	0.196	0.397
Health status^a^	Dummy (1 good; 0 otherwise)	0.759	0.428
Wealth index^b^	Continuous	0.026	1.768

^a^for health status (subjective health) good comprises good and very good and otherwise (bad) encompasses mediocre, bad, and very bad.

^b^ Note: the study constructs the wealth index by running PCA on household assets that include energy source, water source, house construction materials, availability of mosquito nets, and type of the housing facility.

In terms of socioeconomic background, we include age of the respondent and the wealth index of their household as continuous variables. The wealth index is constructed by running a Principal Components Analysis (PCA) on household asset dummies (1 capturing those that possess an item and 0 otherwise) that include access to different energy sources (electricity and gas), water source (public network, tanks, bottled, filters and a well), house construction materials (concrete), availability of mosquito nets, and type of housing facility (villa house). Gender was coded as a dummy variable, with 1 for male and 0 for female. Marital status was captured as dummy variable, with a value of 1 for married and 0 for unmarried (including never-married, single, widowed and divorced). Nationality was coded as a binary variable, with 1 for Saudi and 0 for non-Saudi (expatriate). Household income is used as a continuous variable. With regard education level, it was grouped into five categories and was coded as follows: 1 for below primary school (those who can just read and write and the illiterate), and 0 for otherwise; 1 for primary school education and 0 for otherwise, 1 for intermediate school and 0 for otherwise, 1 for secondary school and 0 for otherwise, 1 for higher education (including those with university degree or postgraduate degree) and 0 for otherwise. We also include health status (subjective health) with 1 for those who perceive themselves in a good health (good and very good) and 0 for those who do not think they are in a good health status (bad, very bad and mediocre).

### Empirical Strategy

This study examines how health insurance relates to OOP expenditure on health and on medicines along different levels of income. We therefore build econometric models that specify OOP as a function of insurance, income, and the interaction between insurance and income. The model can be presented as follows.

$$InY_{ij}=\beta_{0}+\beta_{1}Insurance_{i}+\beta_{2}\left(Insurance_{i}\times lnincome_{j}\right)+\beta_{3}lncome_{j}+\beta_{4}X_{ij}+\varepsilon_{ij}$$ (1)

In Eq. 1, Y is the outcome representing either OOP expenditure on health or OOP expenditure on medicines for individual i in household j *.* Eq. 1 captures the average effects of insurance on OOP by $\beta_{1}$ assuming that $\beta_{2}$ is insignificant. A positive $\beta_{1}$ coefficient would imply that insurance increases OOP while a negative coefficient shows a reduction in OOP due to the insurance. Controlling for income (through the log of income variable and the $\beta_{3}$ coefficient), $\beta_{2}$ captures the difference in OOP due to insurance along income. Income is logged in this study for two reasons. First is to ensure that it is normally distributed because income is always skewed to the right in survey data which was also the case in our sample. Second, logging income together with OOP allowed us to interpret the results of the study as direct percentage changes. The average effects of insurance on the gross value of OOP are then equal to $\left(\beta_{1}+\beta_{2}\right)$.

If insurance induces equity in healthcare access signaled by a homogenous reduction in spending, $\beta_{2}$ will be insignificant. Alternatively, if insurance leads to income gaps in healthcare access $\beta_{2}$ will be significant. Precisely, a negative sign could imply that within the sub-sample of the insured OOP reduces with increased income. Thus, the insurance package is comprehensive enough that even the rich reduce out of pocket-payments for sophisticated care. A positive sign could entail that insurance increases awareness of self-health (that can also result from supplier induced demand), which however, is not fully covered by the package. The rich use this knowledge to procure additional healthcare, increasing the healthcare access gap between them and the poor.

In Eq. 1, we also include other OOP covariates, to reduce the effects of omitted variables biasing the relationship of interest. At individual level we control for age, gender, marital status, and the level of education. At household level we include wealth index, which is a summary of the assets acquired by a household in which an individual resides. We capture the error term of Eq. 1 with $\varepsilon_{ij}$.

### Functional Form

Notably, the anticipated effects of health insurance are conditional on the individual incurring OOP expenditure. In healthcare systems that also provide free healthcare services, some people may incur no OOP. Modeling the relationship between insurance and OOP without accounting for the zero OOP expenditures leads to corner solutions unless the estimator adopts an appropriate functional form. We, therefore, estimate Eq. 1 using the Tobit model censored at zero, to evade the corner solutions. An additional concern is about the systematic differences between the insured and the uninsured which we minimize using the Inverse Propensity Scores Weighting technique.

### Inverse Propensity Scores Weighting

The variable health insurance is potentially endogenous since characteristics that affect participation in insurance could simultaneously relate to OOP. An example is the case of adverse selection. Sick people incurring high OOPs may self-select into insurance. This could over- or underestimate the true effects of insurance on OOP. Since some of these confounding factors are unobservable to a researcher, the estimates could be biased. Formally, this would entail that the error term in Eq. 1 correlates with the coefficient of insurance. We minimize these confounding effects by weighting our Tobit estimations with inverse propensity scores.

To construct the weights, we first estimate a logit model of insurance participation as follows:

$$Insurance_{ij}=\alpha_{0}+\alpha_{1}M_{ij}+\mu_{ij}$$ (2)

The explanatory variables, $M_{ij}$, in Eq. 2 are those used is Eq. 1 except the income. ${\text{μ}}_{\text{ij }}$ is the error term. We generate propensity scores of insurance participation conditional on observed characteristics using estimates presented in Table 2. We follow previous literature (Hirano and Imbens, 2001) to weight each observation in the treatment group by 1 and those in the control group by a fraction of 1 minus the propensity score as follows:

$$w_{i}=\frac{\hat{p}\left(M_{i}\right)}{\left(1-\hat{p}\left(M_{i}\right)\right)},\;\;0<\hat{p}\left(M_{i}\right)<1$$ (3)

The Inverse Propensity Scores Weighting (IPW) reduces confounding effects by shifting the distribution of the untreated group’s covariates to match that of the treated. The weights, ${\text{w}}_{\text{i }}$, create a sample where the distribution of covariates is independent of the treatment status.

**TABLE 2:** Marginal effects from logit estimates on factors that affect participation in insurance.

	(1)	(2)
	Insured	Standard errors
Saudi national	−0.583***	[0.015]
Age	0.002***	[0.000]
Male	0.081***	[0.011]
Married	0.087***	[0.014]
Primary school	0.095***	[0.021]
Intermediate school	0.071***	[0.020]
Secondary school	0.127***	[0.019]
Higher education	0.146***	[0.021]
Health status	0.036**	[0.015]
Wealth index	0.060***	[0.003]
Pseudo-R^2^	0.329	
Observations	8655	

Significance levels: *** *p* <0.01, ** *p* <0.05, * *p* <0.1. Standards errors in parentheses.

## Results

### Inverse Propensity Scores Weighting Results for Insurance Participation

Although our interest is to understand the heterogeneous effects of health insurance on OOP expenditure, we begin by validating our methods. Table 2 presents the marginal effects of selection into health insurance from a logit model. The chances of obtaining insurance reduces for non-citizens. This is consistent with the notion that the Saudi health insurance is aimed at covering private sector workers, who are mostly expatriates. Being married, male, educated, and having a good self-assessed healthy status, increases the probability of obtaining insurance. Notably, relatively rich households obtain insurance in comparison to the less well off. The salient picture is that all the included covariates significantly relate to selectivity into health insurance.

**FIGURE 2:**
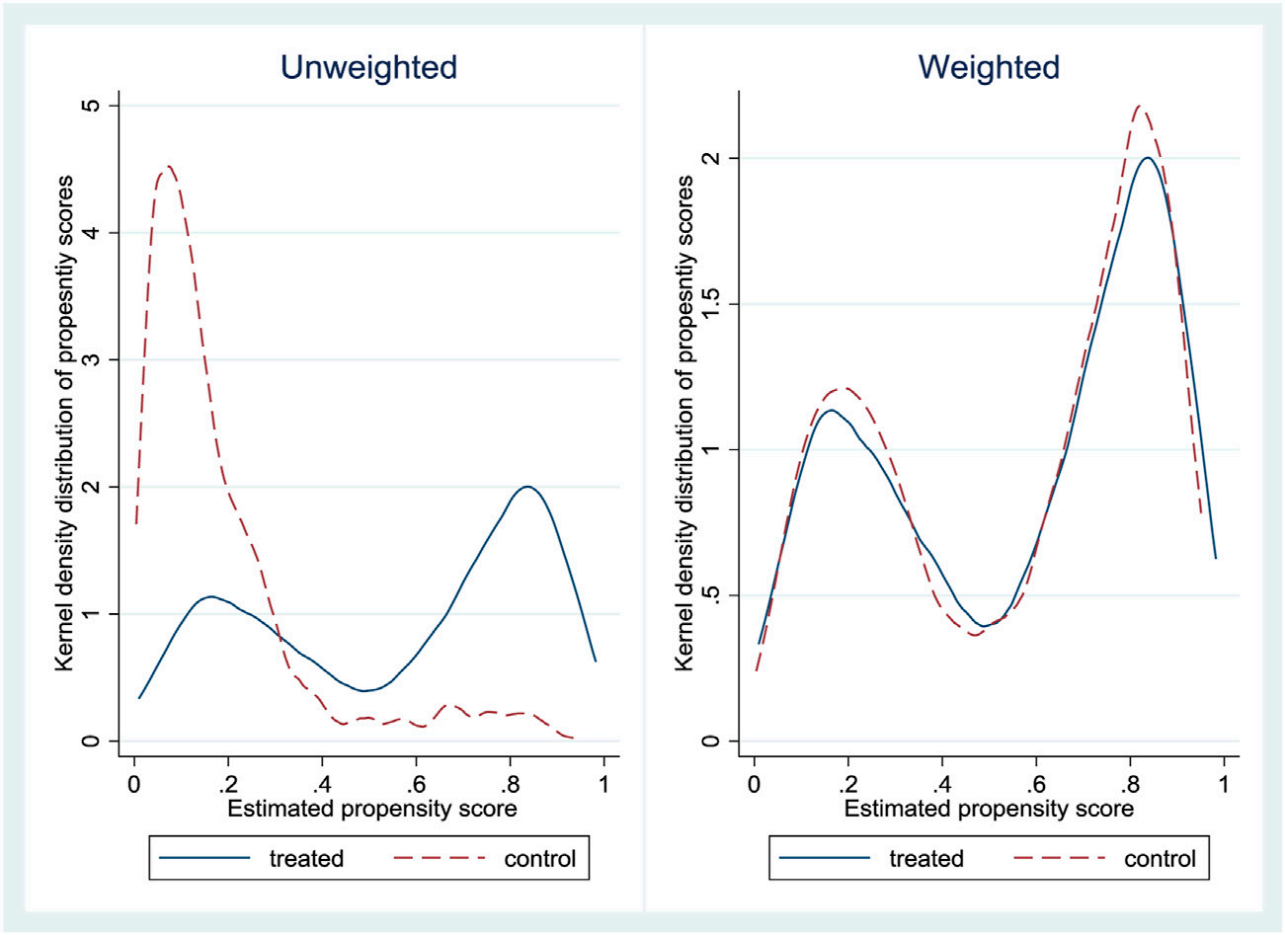
Kernel density distribution of propensity scores for the insured (treated) and uninsured (control).

We then predicted propensity scores of the participation from the logit estimates and compute inverse propensity weights. Figure 2 shows kernel density distribution of the OOP covariates among the treated group of the insured (bold line) and the control group of the uninsured (dotted line). The first plot (left panel) shows the unweighted distribution of the covariates and the second plot (right panel) displays the distribution of covariates that is weighted by the IPW. In the unweighted panel, we observe that the treatment and control groups overlap imperfectly, while the weighted panel reveals more unified distribution of covariates. The IPW pools the distribution of the treated and untreated covariates into more comparable groups and reduces selectivity into insurance bias on observable attributes.

### Descriptive Statistics

Table 3 presents the equality of means, using t-tests, between the insured and the uninsured when using the IPW and when not using the weights. The table also includes income, OOP expenditure on health and OOP expenditure on medicine. Columns 1 to 3 present unweighted results. The insured have high average income while they spend less on OOP expenditure on health relative to the uninsured. This provides a preliminary picture that insurance associates with reduced OOP expenditure on health. The two groups do not differ on medicine related OOP. All covariates remain statistically significant and maintain the signs observed in the insurance participation equation results that are in Table 2. Columns 4 to 6 of Table 3 show weighted differences. Income and OOP expenditure on health remain high and low respectively for the insured relative to the uninsured. Weighted results show that insured individuals spend, significantly, more on medicine OOP relative to the uninsured. Amongst the covariates of OOP only male (positive), those with below primary school education (positive) and individuals with higher education (positive) remain significant after weighting. The loss in significance for the majority covariates, after weighting, emphasizes the reduction in bias on observable attributes by the IPW.

**TABLE 3:** Differences in means between the insured and the uninsured samples.

	Weighted			Unweighted		
	(1)	(2)	(3)	(4)	(5)	(6)
	Insured	Uninsured	*t*-test	Insured	Uninsured	*t*-test
Income	12,308.140	11,077.910	1230.230***	12,308.140	10,387.100	1921.038***
Health OOP	609.900	805.090	−195.190***	609.900	693.110	−83.208**
Medicine OOP	404.850	389.570	15.286	404.850	350.270	54.582*
Saudi national	0.400	0.930	−0.523***	0.400	0.410	−0.009
Age	38.220	35.790	2.424***	38.220	39.450	−1.232
Male	0.610	0.490	0.122***	0.610	0.540	0.069***
Married	0.710	0.540	0.175***	0.710	0.690	0.021
Below primary school	0.232	0.125	0.108***	0.147	0.125	0.022*
Primary school	0.145	0.124	0.021**	0.145	0.124	0.021
Intermediate school	0.153	0.167	0.0138	0.181	0.167	0.014
Secondary school	0.305	0.315	0.0098	0.319	0.315	0.005
Higher education	0.270	0.165	0.105	0.270	0.208	0.062***
Health status	0.840	0.720	0.120	0.840	0.840	0.007
Wealth index^a^	0.470	−0.160	0.628***	0.470	0.440	0.025
Observations	2575	6080		2575	6080	

Significance levels: *** *p* <0.01, ** *p* <0.05, * *p* <0.1.

^a^ The wealth index reveals that insured individuals are wealthier than the uninsured. However, weighting removes the wealth differences between the insured and the uninsured.

In Table A1 of appendix, we examine the differences in OOP expenditure split by income categories and nationality. We also include means, standard deviations and 95% confidence intervals. The sample is first disaggregated by median income, which was 8900 SR in the sample. Those above the median incur more OOP on health (1009 SR) than those below the median (485 SR). The trend is the same with OOP on medicine with those above the median spending more (530 SR) than those below median income. Concerning nationality, Saudis spend more on OOP on health (842 SR) than non-Saudis (425 SR). Similarly, for OOP on medicine, Saudis spend more (450 SR) than non-Saudis (206 SR).

### Factors that Affect OOP Expenditure on Health and OOP Expenditure on Medicine

Table 4 presents factors that relate to OOP expenditure on health and OOP expenditure on medicine. The estimates include income, insurance, the interaction between income and insurance and covariates (control variables) of OOP. However, in the interest of brevity we limit the results description to the coefficients of interest (income, insured and insured*income) and exclude the covariates. The first 2 columns present results on OOP expenditure on health. Column 1 shows the homogenous relationship between income and insurance on OOP. Income increases expenditure on OOP. The health insurance reduces OOP. This is the primary expected role of insurance in cushioning participants from unanticipated health spending. Column 2 includes the interaction between income and insurance. Income maintains a positive relationship with OOP, and insurance remains negatively related to OOP. The interaction between income and insurance is positive and significant. The result reveals that within the subsample of the insured, income increases OOP.

**TABLE 4:** Marginal effects of factors that affect OOP expenditure.

Variable	(1)	(2)	(3)	(4)
	OOP-Health	OOP-Health	OOP-Medicine	OOP-Medicine
Income (log)	0.537***	0.443***	0.572***	0.457***
	(0.031)	(0.047)	(0.030)	(0.042)
Insured	−0.448***	−2.036***	−0.408***	−2.357***
	(0.043)	(0.441)	(0.039)	(0.438)
Insuredincome (log)		0.178***		0.218***
		(0.048)		(0.048)
Saudi national	0.160***	0.175***	0.166***	0.182***
	(0.048)	(0.048)	(0.041)	(0.041)
Age	0.002	0.002	0.005***	0.005***
	(0.002)	(0.002)	(0.002)	(0.002)
Male	−0.204***	−0.201***	−0.139***	−0.134***
	(0.039)	(0.040)	(0.036)	(0.036)
Married	−0.120**	−0.112**	−0.155***	−0.145***
	(0.049)	(0.049)	(0.046)	(0.046)
Primary school	0.163**	0.158**	0.015	0.009
	(0.066)	(0.066)	(0.065)	(0.066)
Intermediate school	0.151***	0.143***	−0.082	−0.092
	(0.055)	(0.055)	(0.057)	(0.056)
Secondary school	0.029	0.024	-0.087	-0.090
	(0.057)	(0.057)	(0.056)	(0.056)
Higher education	−0.050	−0.054	−0.198**	−0.204***
	(0.091)	(0.091)	(0.078)	(0.078)
Health status	−0.212***	−0.185***	−0.158***	−0.126***
	(0.046)	(0.049)	(0.047)	(0.047)
Wealth index	0.018	0.010	0.029	0.018
	(0.012)	(0.012)	(0.012)	(0.012)
Pseudo *R* ^2^	0.0905	0.0927	0.0994	0.1021
Pseudo-log likelihood	−7165.184	−7148.340	−6935.431	−6913.946
Observations	8,655	8,655	8,490	8,490

Note: Standard errors in parentheses. Significance levels: ***p <0.01, **p <0.05, *p <0.1

Column 3 of Table 4 presents results of the homogeneous relationship between income, insurance, and OOP on medicine. OOP increases with income. The coefficient is larger (0.572) compared to the general insurance in column 1 (0.537). Insurance reduced OOP but with a coefficient (0.408), that is less than that for the OOP expenditure on health (0.448). The result shows that medicine is the largest driver of OOP expenditure on health. Column 4 presents results that include the interaction between income and insurance. The findings show that in a subsample of the insured income increases OOP. The coefficient of interaction is larger (0.218) for the OOP on medicine compared to that of OOP expenditure on health in column 2 (0.178). The interacted results for both OOP expenditure on health and OOP on medicine together reveal that the increase in OOP that is driven by insurance centers on medication. The results show that among the insured, high income increases the chance that an individual meets the costs of the increased health care demand. Thus, partial insurance coverage fails to completely offset inequality in healthcare access.

### Robustness Checks: Relationship Between Insurance and OOP Expenditures by Nationality

Saudi Arabia presents a unique case of the healthcare provision. The kingdom provides free access to healthcare to citizens through public healthcare facilities but not for expatriate workers. At the same time, the KSA has one of the largest numbers of expatriate workers. The expatriates rely on OOP and health insurance. Therefore, the aggregated results presented above may mask heterogeneous information by nationality. The expectation is that insurance should be relevant to the expatriates who have limited healthcare alternatives. If insurance is relevant for the citizens only, then the public free healthcare system is less effective in providing the demanded health care to its citizens.

Table 5 presents the relationship between health insurance and OOP expenditure on health split by nationality. Across all models, income relates positively to OOP. Columns 1 and 2 present estimates for the Saudi citizens and show that OOP reduces with insurance. Consistent with the aggregated outcomes, insurance interacted with income increases OOP. Columns 3 and 4 show results for the sub-sample of expatriate workers. While insurance maintains to reduce OOP expenditure on health, there is no relationship between OOP and the interaction between insurance and income. Table 6 repeats these results using OOP on medicine. The findings remain consistent with those of OOP expenditure on health.

**TABLE 5:** Marginal effects of the relationship between health insurance and OOP expenditure on health.

	(1)	(2)	(3)	(4)
Nationality	Saudi	Saudi	Expatriates	Expatriates
Variable	OOP	OOP	OOP	OOP
Income (log)	0.595***	0.402***	0.477***	0.440***
	(0.035)	(0.032)	(0.039)	(0.072)
Insured	−0.347***	−3.630***	−0.487***	−1.093
	(0.039)	(0.550)	(0.074)	(0.756)
Insured*income		0.350***		0.071
		(0.059)		(0.090)
Age	0.007***	0.007***	−0.001	−0.001
	(0.001)	(0.001)	(0.003)	(0.003)
Male	−0.026	−0.036	−0.341***	−0.346***
	(0.041)	(0.041)	(0.064)	(0.064)
Married	−0.102	−0.123***	−0.119*	−0.118
	(0.053)	(0.055)	(0.068)	(0.069)
Primary school	0.236***	0.247***	0.129	0.128
	(0.077)	(0.076)	(0.094)	(0.094)
Intermediate school	0.173***	0.177***	0.149***	0.144*
	(0.068)	(0.068)	(0.074)	(0.075)
Secondary school	0.179***	0.202***	−0.041	−0.043
	(0.065)	(0.065)	(0.078)	(0.078)
Higher education	0.223	0.254	−0.133	−0.140
	(0.071)	(0.072)	(0.131)	(0.134)
Health status	−0.102**	−0.091*	−0.279***	−0.262***
	(0.049)	(0.048)	(0.072)	(0.078)
Wealth index	0.011	0.008	0.015	0.009
	(0.013)	(0.013)	(0.021)	(0.022)
Observations	6,666	6,666	1,989	1,989

Note: Robust standard errors in parentheses. Significance levels: ***p <0.01, **p <0.05, *p <0.1.

**TABLE 6:** Marginal effects of the relationship between health insurance and OOP on medicine.

	(1)	(2)	(3)	(4)
Nationality	Saudi	Saudi	Expatriates	Expatriates
Variable	OOPMED	OOPMED	OOPMED	OOPMED
Income (log)	0.722***	0.430***	0.461***	0.488***
	(0.040)	(0.037)	(0.039)	(0.067)
Insured	−0.242***	−5.188***	−0.517***	−0.075
	(0.040)	(0.613)	(0.063)	(0.678)
Insured*income		0.527***		-0.051
		(0.066)		(0.080)
Age	0.012***	0.012***	0.001	0.001
	(0.001)	(0.001)	(0.002)	(0.002)
Male	−0.062	−0.080	−0.227***	−0.224***
	(0.042)	(0.042)	(0.055)	(0.056)
Married	−0.258***	−0.291***	−0.091	−0.093
	(0.054)	(0.057)	(0.065)	(0.065)
Primary school	0.126	0.145*	−0.012	−0.011
	(0.083)	(0.081)	(0.092)	(0.091)
Intermediate school	0.175**	0.182**	−0.204***	−0.200***
	(0.074)	(0.074)	(0.074)	(0.074)
Secondary school	0.264***	0.303***	−0.250***	−0.249***
	(0.071)	(0.072)	(0.076)	(0.076)
Higher education	0.278***	0.326***	−0.371***	−0.365***
	(0.074)	(0.076)	(0.110)	(0.113)
Health status	−0.186***	−0.170***	−0.110	−0.122*
	(0.053)	(0.052)	(0.071)	(0.074)
Wealth index	0.010	0.005	0.036	0.041**
	(0.015)	(0.014)	(0.020)	(0.020)
Observations	6,563	6,563	1,927	1,927

Note: Robust standard errors in parentheses. Significance levels: ***p <0.01, **p <0.05, *p <0.1.

## Discussion

The results from this study contribute to a long-standing debate on whether health insurance reduces or increases OOP expenditure on health. We model the heterogeneity of the relationship between the insurance and OOP along income to untie this debate. Indeed, our results show that at low levels of income health insurance reduces OOP. Nevertheless, within the insured, income increases OOP. These outcomes support previous studies (Ahmed et al., 2020; Harish et al., 2020; Sriram and Khan, 2020) that established that health insurance reduces health spending through risk pooling. We further clarify that these expenditure-reducing effects of insurance accrue at the lower tail of income distribution. Hence, if the objective of policy is to cushion the relatively poor, then health insurance is a key.

Further, these findings raise a red flag on inefficiencies of healthcare provision induced by possible information asymmetries between healthcare providers and the clients under insurance. Previous evidence shows that health insurance increases health seeking behavior (Jowett et al., 2004; Blanchet et al., 2012; Robyn et al., 2012; Jin et al., 2019). As the insured make more visits to healthcare providers, they discover more about their health and a set of services required to maintain good health. Nevertheless, providers take advantage of the availability of the insured to prescribe care that is not covered by the insurance (Bogg et al., 2016). The choice of what part of the prescription is more essential than the other remains private information of the provider. This encourages supplier-induced demand and rising costs of medical expenditure that one would not have incurred in the absence of insurance.

The result that these rising costs due to health insurance are amongst the relatively rich also highlights persistent inequalities in the quality of and access to healthcare. Thus, if these extra services are essential for good health and they are only attained conditional on high income, then health insurance increases inequalities of access to good health. This is against the anticipated notion that insurance reduces healthcare inequality by making more services accessible to the liquidity constrained, moving them closer to the well-off. Perhaps insurance should be made more generous to cater for the improved demand that it creates if equity in healthcare is to be achieved.

In addition, the results from this study highlighted that free public healthcare systems do not eliminate increased spending on private health services. Particularly, we showed that the relationship between health insurance and OOP and the relationship between health insurance and OOP along different levels of income, are significant amongst people with access to free public healthcare who also happen to be citizens of Saudi Arabia. Free public health care is characterized by several bottle necks such as long waiting lines and limited amount of resources available to provide adequate health care to the population (Alkhamis et al., 2014). Hence, the finding that the heterogenous relationship between health insurance and OOP is only robust amongst Saudi citizens, could be reflecting the significance of these public healthcare provision challenges. Further, we show that these relationships are stronger on medicines relative to OOP expenditure on health. The result could also highlight the possible shortage of medicine in public the healthcare system.

Besides, the relationship between health insurance and OOP expenditure is insignificant amongst expatriate workers in this study. The result could be due to the nature of healthcare access for the expatriates. These workers are not allowed to use free public healthcare services and their employers are mandated, by law, to purchase health insurance for the expatriates to access private healthcare services (Alkhamis, 2018). Unlike public facilities, private facilities are not congested, hence, people have short waiting times. The convenience of using insurance in the private facilities reduces need to opt for quicker but paying services that are already covered by the insurance. Expatriates only pay for services that are not covered by insurance. The absence or presence of insurance should not alter demand for such services. This is confirmed by the positive relationship between income and OOPs by the expatriate workers. Therefore, OOPs amongst expatriates is responsive to income changes but not insurance. Arguably this could be through changes in demand on health care services that are outside the insurance package.

Only a handful of studies have examined the heterogenous effects of health insurance on OOPs. All of them evaluate insurance schemes that were initiated by government as is the case with the KSA scheme. In Mexico (Wirtz et al., 2012), the insurance is found reducing OOPs on medicine with effects of within a range of 1.4–1.7%, that is lower than the 2.2% found in our study. The heterogeneity studied in Mexico is, however, on different types of insurance packages rather than along different levels of income that we examine. Results from China partly support our findings (Wagstaff and Lindelow, 2008). Even though health insurance is found increasing OOP for both the poor and the rich, the increase is more pronounced amongst the rich. The authors argue that the contributory nature of the schemes make high quality services only affordable for the affluent. In Rwanda (Woldemichael et al., 2016), a health insurance is found increasing OOP on outpatient services for the rich while reducing OOP expenditure on health and on medicine. In relation to our findings, the results outside Saudi Arabia reveal that the heterogenous effects of insurance on OOP are context specific: they depend on the coverage of the schemes and income differences between the insured. In the case of KSA that implements a uniform compulsory scheme for non-citizens, OOP generally reduces with insurance, while increasing amongst the rich who are insured.

It is important to note that our study has possible limitations. First, since the data is self-reported it could suffer from recall bias particularly for exact amount of OOP and income. Second, there was a considerable amount of missing information on some health indicators that limited the sample size for analysis. This could be a serious problem where the non-responses are non-random which was not necessarily our case. Third, we only interpret our results as associations not causations recognizing the possibility that selection into insurance could be due to other factors such as risk aversion, that are not observable to a researcher. Fourth, we cannot observe how the heterogeneous relationship between insurance and OOP changes overtime because our data is cross-sectional. However, these findings light up important insights on the direction of association between health insurance and OOP, that it varies conditional on income. Therefore, policy should be aware of the likely implications of promoting health insurance coverage on OOP for people with different income levels; the insurance reduces OOP across the entire population but raises the OOP amongst people with high income (when income increases).

## Conclusion

The study used data from the KSA to show that insurance reduces OOP expenditure on health amongst its participants while increasing the expenditure along rising income amongst them. The results highlight possible supplier-induced demand that insured individuals encounter in setups where insurance coverage is less generous. Further, they show that advanced care remains only accessible subject to income, hence, insurance may not be a panacea for equitable healthcare access. The heterogeneities along income are only robust amongst Saudi citizens (who have free public healthcare access) illuminating the significance of healthcare burdens experienced by free healthcare systems. The relationship between insurance and OOP is stronger on medicine, pointing toward the need for policy to target medicine within the healthcare package if welfare is to register remarkable savings on insurance. Accounting for these heterogeneous relationships between health insurance while also maintaining a robust public healthcare system could therefore go a long way in propelling countries toward attainment of universal healthcare coverage, while being conscious about the potential welfare distribution impacts of the insurance on health spending.

## Data Availability

The datasets generated and/or analyzed during the current study are not publicly available due to privacy, confidentiality, and other restrictions. Access to data can be gained through the General Authority for Statistics in Saudi Arabia. Requests to access these datasets should be directed to Customer support of the General Authority for Statistics, email: cs@stats.gov.sa.

## Appendix

**TABLE A1:** Differences in Out-of-Pocket expenditure by income and nationality.

	Mean	Std Error	95% CI
OOP expenditure on health Below median income Above median income OOP expenditure on Medicine Below median income Above median income			
	485.440	8.006	469.746–501.135
	1009.990	23.800	963.336–1056.644
		
	257.996	4.617	248.946–267.045
	530.956	18.359	494.968–566.944
OOP expenditure on health Non-Saudi national Saudi national OOP expenditure on Medicine Non-Saudi national Saudi national			
	425.829	12.155	402.003–449.655
	842.855	16.089	811.317–874.393
		
	206.659	6.707	193.512–219.806
	450.046	12.161	426.207–473.885

Note: median income in the sample is 8900 Saudi Riyals (Equivalent to US$ 2373,33).
